# Safety and Tolerability of ShigActive™, a *Shigella* spp. Targeting Bacteriophage Preparation, in a Phase 1 Randomized, Double-Blind, Controlled Clinical Trial

**DOI:** 10.3390/antibiotics13090858

**Published:** 2024-09-07

**Authors:** Wilbur H. Chen, Joelle Woolston, Silvia Grant-Beurmann, Courtney K. Robinson, Garima Bansal, Joseph Nkeze, Jasnehta Permala-Booth, Claire M. Fraser, Sharon M. Tennant, Mallory C. Shriver, Marcela F. Pasetti, Yuanyuan Liang, Karen L. Kotloff, Alexander Sulakvelidze, Jennifer A. Schwartz

**Affiliations:** 1 Center for Vaccine Development and Global Health, University of Maryland School of Medicine, Baltimore, MD 21201, USA; 2 Intralytix, Inc., Columbia, MD 21046, USA; 3 Institute for Genome Sciences, University of Maryland School of Medicine, Baltimore, MD 21201, USA; 4 Department of Medicine, University of Maryland School of Medicine, Baltimore, MD 21201, USA; 5 Department of Epidemiology and Public Health, University of Maryland School of Medicine, Baltimore, MD 21201, USA

**Keywords:** bacteriophage, shigella, bacteriophage therapy, alternative antibacterials, microbiome, safety

## Abstract

Bacterial diseases of the gastrointestinal (GI) tract continue to be a major worldwide cause of human morbidity and mortality. Among various enteric pathogens, *Shigella* spp. are some of the most common and deadly bacterial pathogens. They are responsible for ~125 million worldwide cases of shigellosis, and ~14,000 deaths annually, the majority in children under the age of 5 and occurring in developing countries. Preventing and treating shigellosis with conventional drugs (e.g., vaccines and antibiotics) has proven to be very difficult. Here, we assessed the safety and tolerability of ShigActive™, a lytic bacteriophage preparation targeting *Shigella* spp., in a randomized, placebo-controlled, double-blind Phase 1 clinical trial. Ten participants randomized 4:1 received ShigActive™ or placebo co-administered with sodium bicarbonate orally three times daily for 7 days. Solicited and unsolicited adverse events (AEs) were observed for 29 days. Fifty percent of the subjects receiving ShigActive™ reported mild GI-related symptoms, while one participant experienced moderate fatigue. No serious or medically attended AEs occurred through day 90. Additionally, no significant differences in GI-associated inflammatory mediators or fecal microbiome changes were observed between placebo- and ShigActive™-treated subjects, or from a participants’ baseline value. The results of this first-in-human (FIH) randomized, controlled Phase 1 trial of ShigActive™ demonstrate that it is safe and well tolerated when orally administered with no significant differences compared to placebo controls.

## 1. Introduction

Globally an estimated 125 million infections caused by *Shigella* spp. occur each year and are a significant cause of morbidity and mortality, with the majority of the approximate 14,000 annual deaths significantly impacting children under the age of 5 [1,2,3]. In the United States (US), *Shigella* is a leading cause of gastroenteritis, where approximately 500,000 cases of shigellosis occur annually [4,5]. The CDC [6,7] and WHO [8,9] continue to emphasize the threat of antibiotic-resistant *Shigella*, which is underscored further by the increasing prevalence of antibiotic-resistant *Shigella* in food [10], occurrence through sexual transmission [11] and unusually large outbreaks [12]. The increasing prevalence of multi-antibiotic-resistant *Shigella* strains [6,8,10,11] creates the alarming possibility that these already staggering morbidity and mortality rates may further increase.

Preventing and treating shigellosis with conventional drugs (e.g., vaccines and antibiotics) has proven to be very difficult [13,14]. Therefore, alternative approaches for reducing the incidence and severity of *Shigella* infections are urgently needed. Lytic bacteriophages (or phages) present an ideal platform technology for developing a new class of prophylactic and/or therapeutic products for managing shigellosis. Phages potentially offer an attractive complementary tool for managing *Shigella* infections because their mechanisms of action (MOAs) and bacterial resistance differ from those of antibiotics. Thus, antibiotic resistance does not correlate with resistance to phages, and vice versa [15]. In practice, antibiotic-resistant bacteria remain sensitive to, and can be killed by, lytic phages specific for the bacteria. Moreover, recent data suggest in some cases, such as with polysaccharide and drug efflux receptors, that mutations resulting in development of phage resistance in antibiotic-resistant bacteria causes a trade-off in bacterial fitness leading to antibiotic sensitivity [16]. These different MOAs and potential fitness costs provide a significant opportunity for preventing and treating diseases with bacteriophage-based drugs for multi-drug-resistant bacteria, including drug-resistant *Shigella* strains.

Interestingly, the first ever clinical use of bacteriophages also was focused on treating *Shigella* infections (the trial was successfully conducted in France in 1919) [17]. Before antibiotics became available, *Shigella* phages were perhaps the most frequently and successfully used therapies for treating human shigellosis [18,19]. Although thousands of patients have been treated with bacteriophages during the long history of their therapeutic use (since 1919 to present), well-documented, randomized and/or controlled human studies, and formal reporting of adverse events (AEs) per US Food and Drug Administration (FDA) guidelines, have only been carried out in the most recent clinical trials on bacteriophage therapy (reviewed in [20,21,22,23]). In these reviews, the authors conclude, based on their evaluation of published phage therapy case studies and clinical research, that phage therapy is well tolerated. For instance, in Luong et al. (2020), mild AEs only were observed in 12 out of 59 recent cases or studies examined, by all routes of phage administration [21]. Only seven published case studies [24,25,26] or trials [27,28,29,30] evaluating oral phage administration have been recently reported. Of these, just four were clinical trials. Three of these trials were performed in adults [27,28,29], and one included children [30]. Only two of these oral phage therapy trials reported mild AEs [27,28,29].

ShigActive™, a cocktail preparation of five lytic bacteriophages that targets all four species of *Shigella* (*S. boydii*, *S. dysenteriae*, *S. flexneri* and *S. sonnei*), is safe and efficacious against *Shigella* challenge in mice [31]. Here, we report the first-in-human (FIH) Phase 1 clinical trial of ShigActive™ (clinicaltrial.gov: NCT05182749) and the first modern-day randomized control trial (RCT) for an orally administered bacteriophage preparation targeting *Shigella* spp. The primary study objective was to assess the safety and tolerability of ShigActive™ when administered orally to human volunteers (n = 10) at ~1 × 10^10^ plaque-forming units (PFU)/dose, 3 times per day (t.i.d.), for 7 days. Exploratory objectives were: (1) to evaluate the quantity and duration of ShigActive™ in blood and stool; and (2) to determine the impact of ShigActive™ on the fecal microbiome.

## 2. Results

### 2.1. Enrollment and Demographics

**Figure 1:**
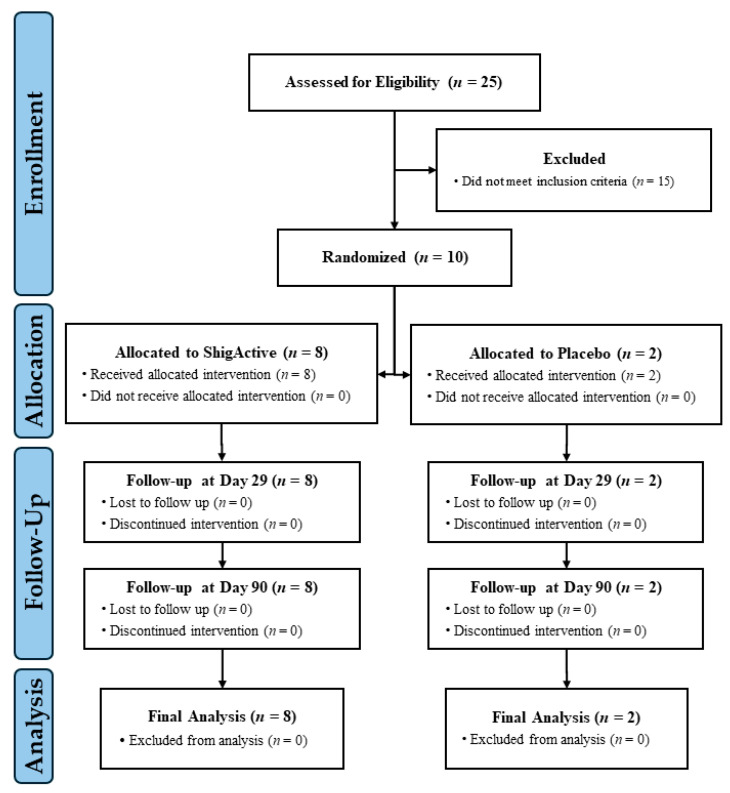
CONSORT diagram.

**Figure 2:**
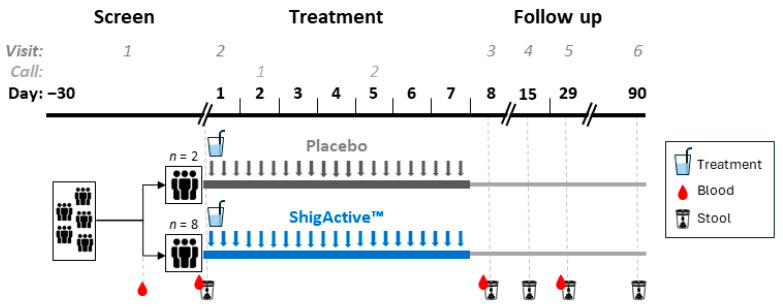
Schematic diagram of study design and procedures.

Ten participants were enrolled in this Phase 1 study on 23 March 2023 (Figure 1). The sample size was selected to rule out unacceptable toxicity before moving to a planned Phase 2a controlled human infection model of shigellosis (clinicaltrial.gov: NCT05182749). The Phase 1 demographics are summarized in Table 1. Half of the participants were male and half female, ranging in age from 23 to 42 years. Of those, 80% were white, 10% Black or African American, and 10% American Indian or Alaska Native. Eight were randomized to receive ShigActive™, while two were randomized to receive the placebo control. Twenty-one self-administered oral doses of ShigActive™ (1 × 10^10^ PFU/dose) or placebo per subject were planned over a 7-day period, approximately every 8 h, with follow-up through day 90 (Figure 2). Several deviations occurred in dosing administration. However, compliance with the total number of planned doses was >90%. Two subjects randomized to ShigActive™ missed either 1 (ID# 15, day 4) or 2 (ID# 24, one each on days 2 and 3) doses over the 7-day treatment period, while one placebo recipient had a single out-of-window dose on day 7.

### 2.2. Safety and Tolerability

Solicited, unsolicited, non-serious and serious or medically attended AEs were assessed (Table 2). A total of 9 AEs, 8 assessed as treatment-emergent, occurred throughout the trial only in the ShigActive™-treated group with 6 solicited (all gastrointestinal [GI]-related) and 3 unsolicited AEs (Table 2). Three out of the 8 ShigActive™-treated participants (37.5%) experienced at least 1 solicited AE during the 7 day treatment period where: 1 subject (ID# 1) experienced mild bloating on days 2 and 8, and reported intermittent excessive gas on day 8; 1 subject (ID# 24) had mild abdominal pain/cramping and mild bloating on day 8; and 1 subject (ID# 8) reported mild excessive gas on day 2. The latter participant reported moderate fatigue on day 4. Two additional mild GI-related unsolicited AEs (i.e., constipation [ID# 24] and diarrhea [ID# 15]) were reported on days 9 and 28, respectively, with the former assessed as not related to treatment. No serious adverse events (SAEs) occurred, and no AEs led to discontinuation of study treatment.

Clinical safety laboratory blood values were evaluated at screening and on days 1, 8 and 29 (Table S1). Four abnormal hemoglobin laboratory values falling just outside of the normal reference ranges were noted across 3 subjects (ID# 14 [placebo], 15, 24) during the study (Table S1). All were deemed not clinically significant, of mild severity, and were observed in both placebo- and ShigActive™-treated participants. These results suggest that orally delivered ShigActive™ is safe and well tolerated.

### 2.3. Pharmacokinectics

As described above, there are only four modern-day reports of RCTs of orally administered phage therapy with limited data on phage pharmacokinetics via the oral route. To assess the pharmacokinetics of ShigActive™, blood and stool were collected throughout the study (Figure 2), and phage levels were quantitated using spot or titration assays. This assay assesses the total *Shigella*-specific PFU activity and does not identify which monophages are present from ShigActive™. No *Shigella*-specific phages were detected at baseline (day 1, prior to first dose) for any sample, none were detected in any blood sample, and none were detected in stool samples from placebo-treated participants. Seven out of eight ShigActive™-treated subjects (87.5%) had detectable levels (i.e., PFU) of *Shigella*-specific phage in their stool on day 8 (Table S2). Of these 7 with detectable phage, the range of total PFU/g of stool was 2.0 × 10^3^ to 5.1 × 10^6^ with a mean of 8.7 × 10^5^ (SD, 1.9 × 10^6^) and a median of 6.1 × 10^4^ (Q1, 5.8 × 10^4^; Q3, 4.6 × 10^5^). Interestingly, one subject (ID# 5) had detectable levels of *Shigella*-specific phage in their stool for the remaining duration of the study on days 15 (3.3 × 10^6^ PFU/g), 29 (4.2 × 10^4^ PFU/g) and 90 (2.2 × 10^4^ PFU/g), and one subject (ID# 22) had no detectable phage at any of the timepoints examined.

**Figure 3:**
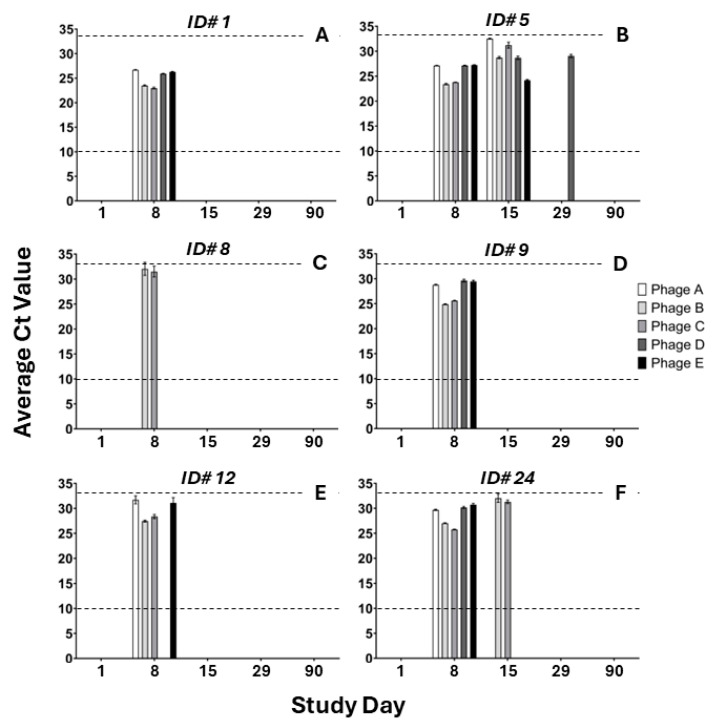
ShigActive™ monophage DNA detected in stool from ShigActive™-treated subjects using qPCR. Shown are the average Ct values for ShigActive™ monophage DNA in stool determined using qPCR performed in triplicate at the indicated timepoints for 6 out of 8 ShigActive™-treated subjects ([**A**] ID#1; [**B**] ID#5; [**C**] ID#8; [**D**] ID#9; [**E**] ID#12; [**F**] ID#24) with detectable levels of monophage DNA (Table S3). Samples were deemed positive for a monophage if the Ct value was >10 and <33, shown as dashed horizontal lines, in at least 2 out of the 3 replicates. Individual monophages (i.e., Phages A–E) are depicted by the shaded bars.

To determine which, if any, monophages from the ShigActive™ cocktail were present, the phage levels in stool extracts also were evaluated using qPCR to detect phage DNA, which can distinguish between each of the five monophages in the ShigActive™ cocktail. As expected, no ShigActive™ monophages were detected at baseline (day 1, prior to first dose) for any sample or in any stool samples from placebo-treated participants (Table S3). Six out of eight ShigActive™-treated subjects (75%) had detectable levels of ShigActive™ monophages in their stool by qPCR on day 8 (Figure 3; Table S3). Two subjects (ID# 5 and 24) had detectable ShigActive™ phage in stool at day 15, while only one subject (ID# 5) had detectable levels at day 29 (Figure 3; Table S3). By qPCR, no ShigActive™ phage was detected in stool at day 90 (Figure 3; Table S3). Two ShigActive™-treated subjects (ID# 15 and 22) had no detectable ShigActive™ phage in stool at any of the timepoints examined by qPCR (Table S3). When the day 1 stool sample of each subject was spiked with ShigActive™ at ~1 × 10^7^ PFU/mL, all ShigActive™ monophages were detected; however, at slightly higher Ct values than the control ShigActive™ at 1 × 10^7^ PFU/mL (Table S3). Five out of the 9 qPCR-positive samples, across all timepoints, detected all 5 monophages from the ShigActive™ cocktail. Four of the 9 qPCR-positive samples detected between 1 and 4 of the 5 ShigActive™ monophages: ID# 12, on day 8, had 4 detectable monophages; ID# 8, on day 8, had 2 detectable monophages; ID# 24, on day 15, had 2 detectable monophages; and ID# 5, on day 29, had 1 detectable monophage (Figure 3, Table S3).

### 2.4. Intestinal Inflammation

**Figure 4:**
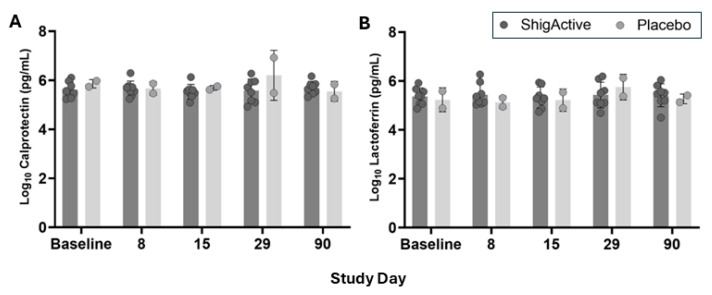
Levels of inflammatory mediators in stool for each treatment group. Shown are the average levels (pg/mL) of (**A**) calprotectin (CP) and (**B**) lactoferrin (LF) in stool at the indicated timepoints for placebo- (light gray bars/symbols) and ShigActive™- (dark gray bars/symbols) treated subjects. Baseline samples are shown prior to the first dose and are from day 1, except for subject ID# 11, whose screening sample was used for baseline due to insufficient stool sample for day 1. Symbols represent the data points for each individual subject at the indicated timepoint. Error bars represent standard deviation.

To assess the potential impact of ShigActive™ on inflammation in the GI tract, levels of the inflammatory mediators calprotectin (CP) and lactoferrin (LF) were measured in stool before, during, and after treatment (Figure 4). No statistically significant differences were observed in fecal CP and LF levels between placebo- and ShigActive™-treated subjects, or from baseline through the end of study (day 90). These results suggest that neither ShigActive™ nor the placebo, both administered with sodium bicarbonate, impact inflammation in the bowel.

### 2.5. Changes in Microbiome Composition

#### 2.5.1. 16S rRNA Gene Sequence Output

Gut microbiota analyses were carried out on 30 fecal samples that represented 10 study participants across 3 timepoints (days 1, 8 and 29). In total, 5836 amplicon sequence variants (ASVs) and 3,052,313 reads were identified in 30 analyzed fecal samples (101,744 average reads per sample). The analysis of the bacterial taxa composition was computed on the filtered dataset (singletons and low prevalent taxa were removed), which yielded 457 ASVs.

#### 2.5.2. Alpha Diversity Did Not Change from Baseline through Day 29

**Figure 5:**
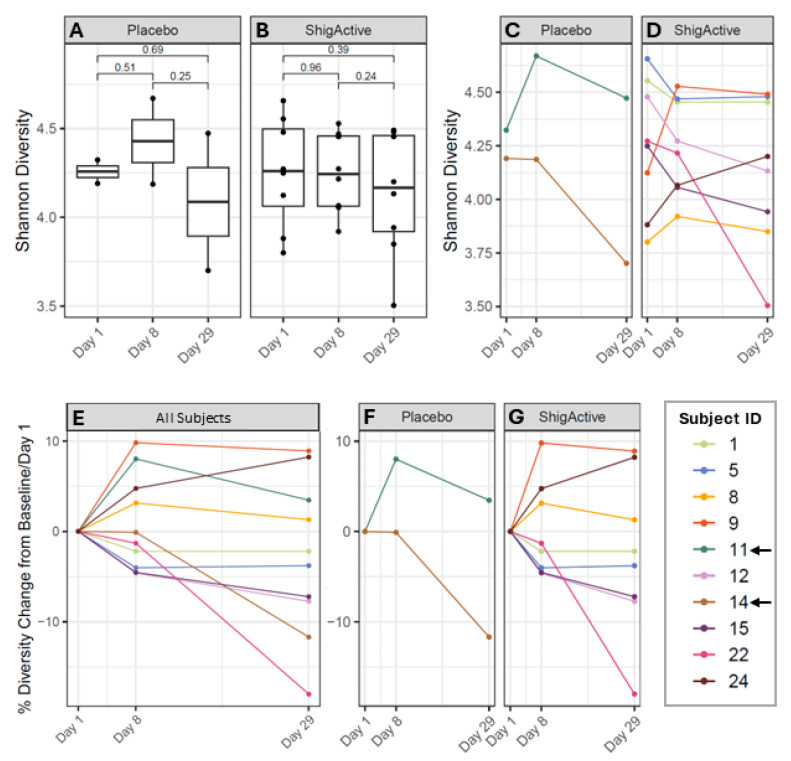
Longitudinal alpha diversity changes for each treatment group. (**A**,**B**) Average Shannon diversity values from baseline/day 1 through day 29 separated by treatment. *p*-values are shown by brackets. (**C**,**D**) Individual Shannon diversity changes at each study timepoint for each participant separated by treatment. Percent Shannon diversity change compared to baseline/day 1 for (**E**) all subjects, or separated by (**F**) placebo or (**G**) ShigActive™ treatment groups. Arrows highlight placebo-treated subjects.

To determine whether the treatment (i.e., placebo vs. ShigActive™) caused any changes in the gut microbiota composition, we compared the Shannon diversity at baseline (day 1, prior to first dose) to the Shannon diversity at the end of the treatment period (day 8) using a *t*-test (Figure 5A–D). We did not observe any significant changes in the Shannon diversity from baseline to day 8 in placebo or ShigActive™ recipients (*t*-test, *p* = 0.51 and *p* = 0.96, respectively). There were also no significant changes from baseline to 3 weeks after treatment ended (day 29) for placebo- or ShigActive™-treated subjects (*t*-test, *p* = 0.69 and *p* = 0.39, respectively), or from day 8 to day 29 (*t*-test, *p* = 0.25 and *p* = 0.24, respectively).

Since each participant exhibited a different baseline Shannon diversity, we blindly calculated the percent Shannon diversity change from baseline/day 1 (prior to first dose). This allowed for easier visualization that there were no longitudinal trends present (Figure 5E–G) where 5 participants exhibited a decreased diversity from baseline to day 8; 4 participants showed an increase; and 1 participant remained at their baseline level. All analyses were initially performed blinded. Upon unblinding, there were no significant percent diversity changes observed for the placebo or ShigActive™ recipients (*t*-test, *p* = 0.26 and *p* = 0.25, respectively). Additionally, incidence of AEs did not appear to correlate with diversity changes as 2 of the 4 (50%) ShigActive™-treated subjects who experienced at least 1 AE had increased changes (Subject IDs #8 and 24, Figure 5D,G), while the other 2 (50%) had decreased changes (Subject IDs #1 and 15, Figure 5D,G).

#### 2.5.3. Variable Microbial Communities Observed among Participants with Greater Shifts at Later Timepoints

**Figure 6:**
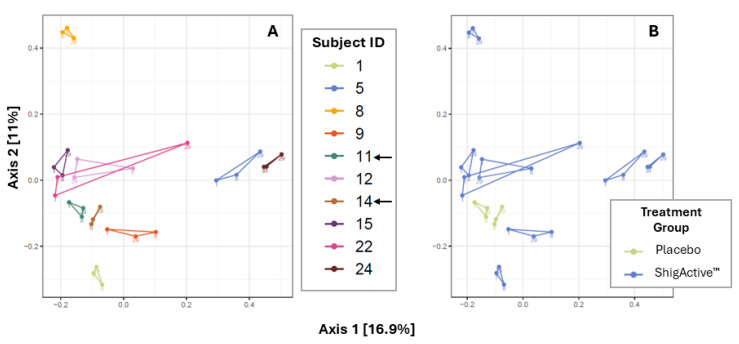
Principal coordinates analysis (PCoA) using Bray-Curtis dissimilarity. Panels show the beta diversity changes through day 29 displayed by (**A**) individual subject and (**B**) treatment group. Arrows highlight placebo-treated subjects.

**Figure 7:**
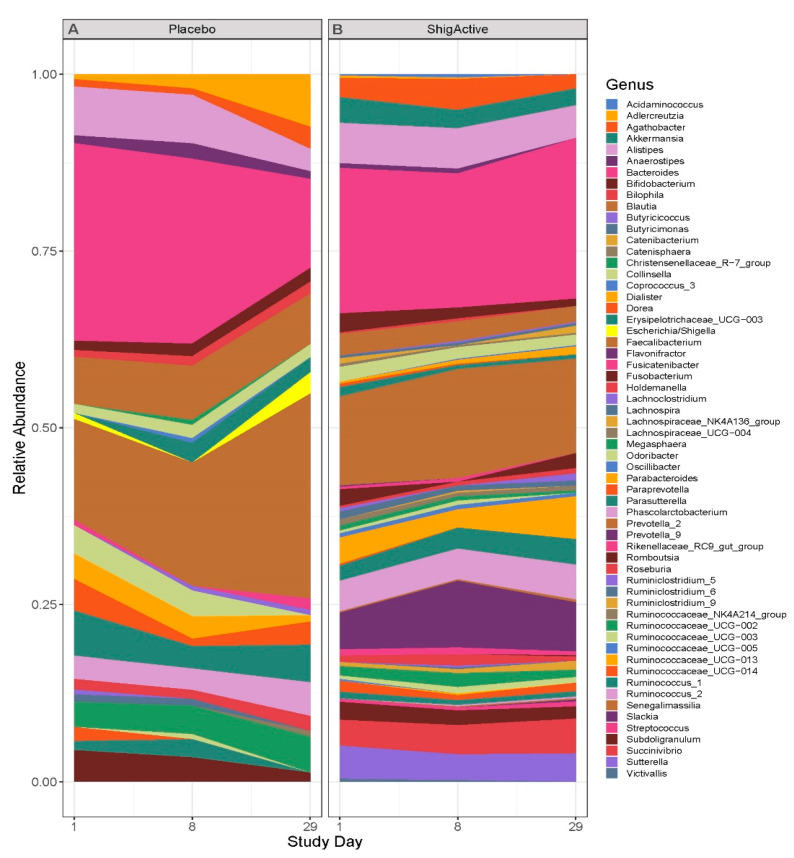
Longitudinal taxonomic differences observed across the two treatment groups through day 29. The average relative abundance for each bacterial genus from day 1 (baseline) through day 29 for the (**A**) placebo- or (**B**) ShigActive™-treated groups. Only genera with relative abundance that are greater than 1% are shown. Relative abundance for each bacterial genus graphed by individual subject is shown in Figure S1.

**Figure 8:**
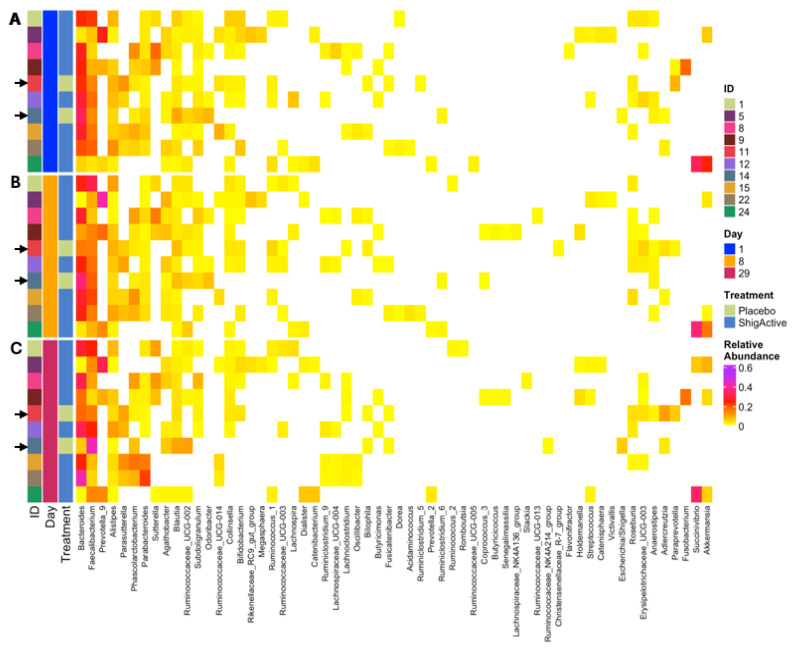
Heatmap displaying the taxonomic distributions using relative abundances. Each study day was plotted separately ((**A**), day 1/baseline [blue]; (**B**), day 8 [orange]; (**C**), day 29 [maroon]), with each sample shown along the *y*-axis. The participant IDs (column 1), study day (column 2), and treatment assignment (column 3) are marked on the left side of the heatmap. Arrows highlight placebo-treated subjects. Only genera with relative abundance that are greater than 1% are shown.

Principal coordinates analysis (PCoA) was performed to visualize the beta diversity using the Bray-Curtis dissimilarity method, and permutational multivariate analysis of variance (PERMANOVA) was used to detect significant microbial community structure differences between the study timepoints (Figure 6). Treatment and study day exhibited a significant effect on the variance of the community composition (PERMANOVA, *p* = 0.013 and *p* = 0.011, respectively). The community composition change was smaller from baseline/day 1 to day 8 than from day 8 to day 29 for most participants (with the exception of participants ID# 1, 9, 11 [placebo-treated], and 12). While more inter- than intra-variability of the participants’ gut microbiota composition was observed, no significant taxonomic changes occurred between study timepoints (Figure 7, Figure 8 and Figure S1).

Assessment of community composition excluded taxa with less than 1% relative abundance. Among those excluded were the *Escherichia*/*Shigella* genera, which are of particular interest here and were excluded in the analyses for all subjects except for placebo-treated subject ID# 14 (Figure 8 and Figure S1). At baseline (day 1, prior to first treatment dose), stool samples from both placebo-treated subjects (100%) and 6 out of 8 ShigActive™-treated subjects (75%) had detectable, but less than 1%, relative abundance of the *Escherichia*/*Shigella* genera (Table S4). These levels varied from day 1 through day 29 for the placebo-treated subjects. In the ShigActive™-treated subjects with detectable levels at baseline, relative abundance of the *Escherichia*/*Shigella* decreased by day 29 in 4 out of the 6 subjects (66.7%; of which 3 had undetectable levels by day 29), while in the remaining 2 subjects the levels remained relatively unchanged (Table S4).

## 3. Discussion

The first use of phage therapy occurred in the early 1900s. However, with the rise of antibiotics, phage therapy fell out of practice in the West, although use continued in Eastern Europe and in countries of the former Soviet Union. The rise and threat of antibiotic resistance has renewed interest in phages as an alternative or adjunct therapy to treat bacterial infections (reviewed in [19]). Yet, despite over 100 years of phage therapy practice, significant gaps remain [23], specifically in the performance of RCTs that may shed light on gaps in scientific and practical knowledge. Here, we take a step forward towards a phage-based therapy to reduce and/or eliminate the severity and/or incidence of shigellosis, a significant public health concern.

In this work, we investigated the safety and tolerability of ShigActive™ in an FIH Phase 1 RCT. The most salient findings of our study are that a 7 day t.i.d. course of orally administered ShigActive™ (1) was safe and well tolerated with mostly only mild symptoms reported (Table 2 and Table S1); (2) resulted in detectable levels of phage in stool at the end of the treatment period (Figure 3 and Tables S2 and S3); (3) did not induce fecal markers of GI inflammation (Figure 4); and (4) did not induce significant compositional shifts in the gut microbiome (Figure 5, Figure 6, Figure 7, Figure 8 and Figure S1).

Consistent with a long history of safety with phage therapy interventions (reviewed in [19]), ShigActive™ was well tolerated with no occurrences of SAEs or medically attended AEs. Therefore, AEs that may occur over a rate of 37.5% or higher can be ruled out. Almost all symptoms reported by ShigActive™ recipients were mild and GI-related, with 4 out of 8 ShigActive™-treated subjects (50%) reporting at least one AE. Only one subject in this group reported moderate fatigue during the treatment period. No AEs were reported by the 2 placebo controls. Both the ShigActive™ and placebo doses were preceded by and co-administered with sodium bicarbonate to neutralize the pH of the stomach to prevent acid degradation of the ShigActive™ phages [32,33], allowing for passage of viable phage into the intestines, the intended site of pharmacologic activity for reducing and/or preventing shigellosis. Sodium bicarbonate consumption is known to cause GI-related symptoms, including bloating, excessive gas and abdominal pain [34]. These side effects are attributed to the generation of carbon dioxide when the sodium bicarbonate reacts with gastric fluids [34,35,36]. Therefore, while the two placebo recipients did not report any AEs, we cannot rule out that the treatment-emergent AEs in the ShigActive™-treated subjects was not caused or confounded by sodium bicarbonate due to the small placebo sample size.

Additionally, other clinical assessments support the safety profile of ShigActive™. Vital signs and physical assessments were as expected for all participants in this generally healthy population. Some clinical laboratory values were marginally outside of the normal reference ranges in 5 subjects (2 placebo and 3 ShigActive™ recipients; Table S1). In each case, the associated baseline value was just within the normal reference range and were therefore determined by the Clinical Investigator to be mild and not of clinical significance.

To assess the pharmacokinetics of ShigActive™, *Shigella*-specific phages were quantitated in blood and stool before and after treatment using spot assays for viable phage. As expected, no *Shigella*-specific phages were detected at baseline or in placebo controls in any sample. All but one ShigActive™-treated subject had detectable phage in stool (assessed either by spot assay or qPCR) after the 7 day t.i.d. treatment regimen. This is consistent with findings from other orally administered phage therapy RCTs [27,37,38,39], some of which showed persistence of fecal phage levels in subjects out to at least 3 to 17 days after the last phage dose [27,37,38]. Interestingly, one subject (ID# 5) had persistent levels of phage through the last study day (i.e., day 90).

The spot assay method used here does not distinguish whether the *Shigella*-specific phage detected in stool originated from the ShigActive™ cocktail consisting of 5 monophages or which monophage is present. To address this, the stool samples were also examined to assess the presence of DNA from each of the individual monophages in ShigActive™. In six of the eight ShigActive™-treated subjects, at least two of the five ShigActive™ component monophages were detected, with the majority having all five ShigActive™ monophages at day 8. While the spot assays and qPCR were consistent in demonstrating which 6 ShigActive™-treated subjects shed *Shigella*-specific phage after treatment, there was slight variability between the 2 assays in how long phage may have persisted after treatment ended. As the qPCR only detects for the presence of DNA, while the plating assay detects viable phages, conclusions cannot be drawn about whether this correlates with viable phage persistence that was below the level of detection in the spot assay.

The persistence of the viable phage in one subject (ID# 5), with an increase in viable phage between day 8 and day 15, after phage dosing had ended, indicates the presence of a susceptible strain in this individual. ShigActive™ previously has been shown to lyse strains of the closely related species *E. coli* and *Salmonella* [31]. Subject ID# 5 could potentially harbor a small population of commensal bacteria able to support ShigActive™ phage propagation, such as *E. coli*, that persisted in the gut mucosa but was not represented in the stool samples. The microbiome analysis of this subject showed that the abundance levels for *Escherichia*/*Shigella* decreased over time.

In contrast to stool, *Shigella*-specific phages were not detected in any blood sample. Oral administration of phage previously has been shown to be a poor method of systemic delivery [40]. Interpretation of published data evaluating translocation from the gut to blood is compounded by limitations in understanding phage pharmacokinetics and gut translocation, and by the impact of different variables on phages within the host, such as the potential for each phage to have different properties (e.g., stability) under similar conditions, and sequestration of phage by innate immune responses prior to transit to blood (e.g., IgA binding, transit to intestinal lymph nodes) [40]. Other variables include differing stability profiles of individual monophages in blood and/or plasma [41], dilution in blood and limits of detection in phage quantification [40]. In this study, no phage was detected in blood on day 8 (i.e., day after the last dose), suggesting that ShigActive™ phages did not translocate from the gut to blood. However, additional analyses in future studies would be advised to rule out other potential pharmacokinetic mechanisms (e.g., kinetics of stability in blood and/or plasma) that may alter this interpretation.

Phages have high specificity for their bacterial host and are believed to have minimal impact on human and animal subjects. Here, we explored whether ShigActive™ could induce inflammation or compositional shifts in the microbiome in the gut. Fecal CP and LF are biomarkers of GI inflammation and are recommended testing in chronic diarrhea [42,43,44,45]. Unwarranted induction of inflammatory mediators is of particular interest as ShigActive™ is intended to treat and/or prevent bacillary dysentery [43]. Levels of CP and LF were evaluated in stool throughout the duration of the trial. No significant differences in fecal CP and fecal LF were observed between the placebo- and ShigActive™-treated groups, nor from baseline through the end of the study for each individual subject.

To further elucidate whether ShigActive™ has an impact on the microbial composition in the gut, stool samples collected from baseline (day 1, prior to first dose) through day 29 (~3 week after the last dose) were subjected to 16S rRNA metagenomic analysis. A limited number of RCTs have reported gut microbiome data after oral phage administration, two in healthy volunteers [37,39] and one in children with acute diarrhea [38]. Here, we show that ShigActive™ did not induce significant compositional shifts in the gut microbiome. No differences in alpha diversity were observed between the two treatment groups or from baseline after phage treatment. Additionally, no significant taxonomic changes were observed between timepoints in each subject. Beta diversity changes were observed; however, comparative analysis of taxa enrichment between placebo- and ShigActive™-treated subjects was hindered by the small placebo group size and high variability between the two placebo recipients. Taken together, the biomarker and microbiome data demonstrate no apparent impact of ShigActive™ on markers of gut health, further supporting the gentle and highly specific nature of phage therapy.

Despite the limited sample size, our study design is suitable for the assessment of unacceptable toxicity, high frequency AEs and preliminary analyses of impact on host markers. In conclusion, the results of this FIH Phase 1 trial show that ShigActive™ can be delivered to and bioavailable within the GI tract without apparent induction of inflammation in a safe and well-tolerated manner. This is consistent with the long history of phage therapy safety and begins to bring phage therapy full circle with the first clinical use of phages to treat *Shigella* in 1919 [17].

## 4. Materials and Methods

### 4.1. Trial Design and Participants

A double-blinded, placebo-controlled, randomized Phase 1 clinical trial was performed at a single site at the Center for Vaccine Development at the University of Maryland, Baltimore School of Medicine in Baltimore, MD, USA. Generally healthy male and non-pregnant/non-breastfeeding female participants, aged 18–50 years, meeting the inclusion/exclusion criteria were eligible to enroll. No formal sample size calculations were performed to guide the sample size of the Phase 1 study. The sample size for this study was selected only for the following: (1) to provide an initial FDA-approved clinically monitored proof-of-concept assessment of the safety and tolerability profile of ShigActive™; and, (2) to generate novel, preliminary microbiome data of bacteriophage interaction with normal gut microbiota. The trial is registered, and the eligibility criteria are described on clinicaltrials.gov (NCT05182749). The trial protocol and informed consent were reviewed by the FDA and approved by the Institutional Review Board of the University of Maryland, Baltimore. All participants provided written consent prior to any study procedures or activities.

### 4.2. Interventions and Randomization

ShigActive™ is an aqueous bacteriophage cocktail preparation containing 5 lytic monophages that target all 4 species of *Shigella* (developed and manufactured by Intralytix, Inc., Columbia, MD, USA) [46]. The 5 monophages comprising the cocktail are represented at approximately equal concentrations and have distinct targeting profiles along with overlapping redundancies in killing *Shigella* spp. strains. Ten participants were randomized 4:1 to receive a single dose of ShigActive™ at 1 × 10^10^ PFU/dose or placebo (phosphate-buffered saline [PBS]). Randomization sequence was generated by the study statistician using randomization with random block sizes of 5 or 10 and an assignment ratio of 4:1 for the two study arms. Each product was supplied in identical vials and was indistinguishable from each other in appearance. ShigActive™ or placebo was orally consumed 3 times per day, approximately 8 h apart, for 7 d (21 planned doses total). Prior to each administration (~5 min), participants consumed approximately 120 mL of fresh sodium bicarbonate solution (2 g sodium bicarbonate/150 mL water). ShigActive™ or placebo was co-administered with sodium bicarbonate solution (in ~30 mL) and was prepared immediately prior to consumption.

### 4.3. Study Procedures

In-person visits occurred at screening (within 30 d of day 1) and on days 1, 8, 15, 29 and 90. All day 1 sample collections and measurements were prior to the first dose and were used, unless otherwise indicated, as baseline. Telehealth (by phone or video) visits occurred on days 2 and 5 (Figure 2). Participants were observed for 30 min after the first dose and were followed for 90 d for safety and tolerability. Solicited (i.e., prespecified), unsolicited and serious AEs were recorded and graded daily on electronic diaries by the participant through day 8, and assessed by clinical research personnel on days 15 and 29. The prespecified solicited AEs for days 1 through 7 were the following: diarrhea, fever, vomiting, nausea, abdominal pain/cramping, bloating, tenesmus, anorexia/loss of appetite, excessive gas and headache. Serious adverse events and medically attended AEs were assessed through day 90. Blood was collected for (1) clinical laboratory values (i.e., hematology, biochemistries) at screening, and on days 1, 8 and 29, and (2) phage quantitation on days 1 (baseline), 8 and 29. Stool samples were collected as follows: (1) for quantitation of phage and inflammatory mediators on days 1 (baseline), 8, 15, 29 and 90; and (2) for determination of microbiome composition on day 1 (baseline), 8 and 29. All lab testing and research assays were performed on deidentified samples and blinded to treatment. Vital signs were assessed at each visit. A complete physical exam was performed at screening (within 30 d of day 1), and targeted physical exams were performed at each visit if deemed necessary by the clinical investigator. Urine was assessed for pregnancy testing at screening (within 30 d of day 1) and on day 1 prior to the first dose.

### 4.4. Bacteria

SH.s109, a derivative of *S. sonnei* strain SH.s43 (clinical isolate from Pakistan; original ID# 90; University of Maryland Baltimore) [47], was used as the host strain for total phage quantification as it is susceptible to all 5 monophages comprising ShigActive™. SH.s109 was derived by curing the endogenous plasmid in SH.s43-containing genes for tetracycline, sulfanilamide and streptomycin resistance (*tetA*, *sul2* and *strAB*, respectively) by passage in liquid Luria–Bertani (LB) broth supplemented with a sublethal concentration of mitomycin C (1 μg/mL), followed by single-colony isolation on LB agar. The cured SH.s109 strain was confirmed by gel electrophoresis, PCR and sequencing.

### 4.5. Stool Extracts

Fresh, unpreserved whole stool specimens (1 g), maintained on ice (not frozen), were used for quantification of viable phage by spot assays. Stool samples were mixed with 10 mL of room temperature (RT) SM buffer (Thermo Scientific, Waltman, MA, USA) by vortexing, centrifuged at 1500–2000× *g* for 10 min, and ~3 mL of the suspension was filtered through a 0.22 μm syringe filter. A 100 μL aliquot of the stool extract was combined with 100 μL of excipient (10% monosodium glutamate/1% gelatin) and immediately transferred to −80 °C for phage quantitation using qPCR analysis.

Stool extracts for biomarker analyses were prepared as previously described [43]. Briefly, 300 mg of fresh stool sample was processed in a mini-Beadbeater-8 tissue homogenizer (Biospec, Bartlesville, OK, USA; three 1 min cycles with 2 min incubations on ice between cycles) with ~1.5 g 2.3 mm zirconium beads (Biospec) in 1 mL extraction buffer (PBS [pH 7.4], 0.01% soybean trypsin inhibitor, 0.1% EDTA, 0.5% phenylmethanesulfonyl fluoride solution, and 0.05% Tween 20 [all from Sigma, St. Louis, MO, USA]). After centrifugation (30 min, 4 °C, 14,000 rpm), the supernatant was collected and mixed with 10 μL of 1% bovine serum albumin with 0.1% sodium azide (*v*/*v*; Sigma) and stored at −80 °C for batch analysis.

Stool samples (1 g) for microbiome analysis were placed in OMNIgene-GUT tubes (DNA Genotek, Ottawa, ON, Canada) and stored at −80 °C. Extracts were prepared in batch as described below.

### 4.6. Phage Enumeration

A standard spot test assay using a double agar overlay method was used to quantitate phage in stool [48]. Briefly, to a transfer tube containing 100 μL of host strain, SH.s109 (0.4 ± 0.1 OD_600_), 3 mL of warm (52–54 °C) LB top agar (2.5 g of LB base, 0.7 g of bacteriological agar in 100 mL of ddH_2_O and autoclaved at 15 min at 121 °C) was added and immediately poured on top of LB agar plates and allowed to solidify for 10–30 min at RT. Tenfold serial dilutions of stool extracts were performed using LB broth, of which 10 μL was spotted in duplicate on the top agar plates along with positive (i.e., ShigActive™, ~1 × 10^10^ PFU/mL) and negative (i.e., LB broth) controls. Spots were allowed to dry for 30–60 min at RT, incubated aerobically at 30 °C for 20 ± 2 h and PFUs counted. The detection limit for this assay is ~1 × 10^3^ PFU/g stool.

Stool extracts were further tested to determine which ShigActive™ monophages were detectable in the stool sample. The frozen stool extracts were tested with qPCR assays that targeted each of the five ShigActive™ monophages. Each 20 µL reaction contained 1X iTaq Universal SYBR Green Supermix (Bio-Rad, Hercules, CA, USA), forward and reverse primers (see Table S5) at a final concentration of 500 µM each, and 1 µL of template. The subject samples were run in triplicate against each primer set, along with a positive (i.e., ShigActive™, ~1 × 10^10^ PFU/mL, each component monophage approximately equally represented at ~2 × 10^9^ PFU/mL) and a negative (i.e., water) control included on each plate. The qPCR amplification was run on the QuantStudio 5 Real-Time PCR system (ThermoFisher Scientific, Waltman, MA, USA) with the following conditions: 1 cycle of 95 °C for 3 min; 40 cycles of 95 °C for 20 s and 60 °C for 30 s; melt curve of 60–95 °C. The reactions were determined to be positive if the Ct value was <33 cycles and the peak melting temperature was ±1 °C of the expected melting temperature for that primer set (Table S5). A sample was considered positive for a given primer set if at least 2/3 replicates were positive. To determine if amplification was inhibited by the stool extract, the day 1 extracts were spiked to a final concentration of 1 × 10^7^ PFU/mL ShigActive™ (each component monophage approximately equally represented at 2 × 10^6^ PFU/mL) and tested against all primer sets. The detection limit for this assay is ~1 × 10^3^ PFU/mL when using ShigActive™ alone as the test article.

To determine the amount of phage in blood, a titration assay was used. Blood was collected from each participant into a sodium citrate collection tube and maintained on ice until used fresh in phage enumeration assays. One hundred microliters of each blood sample and host strain, SH.s109 (~0.4 OD_600_), was added to 0.9 mL of RT LB broth, incubated at RT for 10 min, followed by the addition of 3 mL of warm (52–54 °C) LB top agar to the blood–culture mixture. This mixture was then poured on top of LB agar plates, allowed to dry for at least 10 min at RT, incubated aerobically at 30 °C for 20 ± 2 h and PFUs counted. ShigActive™ alone (diluted to 5,000 PFU/mL) and LB broth were used as positive and negative controls, respectively. The detection limit for this assay is ~10 PFU/mL blood.

### 4.7. Inflammatory Mediators

Levels of CP and LF in the stool supernatants were quantified using R-PLEX Human Calprotectin and R-PLEX Human Lactotransferrin Assays, respectively, following the manufacturer’s instructions (Meso Scale Diagnostics [MSD], Rockville, MD, USA). Briefly, 96-well singleplex GOLD 96-well Small Spot Streptavidin SECTOR Plates (MSD) were coated for 1 h with the respective biotinylated capture antibodies prepared to 1X in MSD Diluent 100. All incubations were performed in a RT (20–26 °C) incubator on a plate rotator at 700 rpm. After coating, CP plates were washed with 1X MSD Wash Buffer; LF plates were washed with 1X MSD Tris Wash Buffer. Samples diluted 1:25 in Diluent 101 were added to CP plates. For LF, samples were diluted to 1:500 in Diluent 100. Calibrators (MSD Human Lactotransferrin Calibrator in Diluent 100 or MSD Human Calprotectin Calibrator in Diluent 101) were prepared, and then 4-fold serially diluted. Diluted samples, calibrators, and diluent-only blanks were added in duplicate to assay plates. Plates were incubated for 1 h and then washed as described above. Bound CP or LF was detected using SULFO-TAG anti-Human Calprotectin or Lactotransferrin Antibodies (MSD) diluted to 1X in Diluent 37 or Diluent 100, respectively. After 1 h incubation, plates were washed once more. GOLD Read Buffer B (MSD) was added to all wells of all plates, and then plates were immediately read on the MESO QuickPlex SQ 120 (MSD). Raw data (electrochemiluminescent [ECL] signals) were captured with Methodical Mind (MSD) software and analyzed by four-parameter logistics (4PL) regression with Discovery Workbench v4.0 (MSD). The 7-point calibration curves, CP or LF, were used to interpolate CP and LF concentrations in the stool samples.

### 4.8. Microbiome Analyses

#### 4.8.1. Microbial DNA Extraction and 16S rRNA Gene Sequencing

Genomic DNA was extracted from stool samples with the MagAttract microbial DNA kit (Qiagen, Germantown, MD, USA) using a custom automated protocol on the Hamilton Microlab Star (Hamilton, Reno, NV, USA). Samples were thawed on ice, and a 200 μL aliquot from the stool sample was used as input for the kit following the manufacturer’s protocol. Cells were lysed by bead beating on the TissueLyser (Qiagen) at 20 Hz for 20 min and the final elution volume was 110 μL. The V3–V4 regions of the 16S rRNA gene were amplified by two-step PCR, with amplicon pooling, sequencing on an Illumina HiSeq 2500 instrument (Illumina, Inc., San Diego, CA, USA), and sequence data processing as previously described [49].

#### 4.8.2. 16S rRNA Gene Sequence Analysis

Amplicon sequence variants (ASVs) generated by DADA2 were taxonomically classified using the RDP Naïve Bayesian Classifier [50] trained with the SILVA v128 16S rRNA gene database [51] as implemented in the dada2 R package [52]. Negative controls generated a negligible amount of sequencing reads, whereas the positive controls generated the expected mock community [53]. The R packages ggplot2 and ComplexHeatmap were used to compute the visualizations of the relative taxa abundances.

### 4.9. Statistical Analyses

#### 4.9.1. Demographics and Safety Analyses

Descriptive statistics were used to characterize the demographic profiles and AEs for all participants and for each treatment group separately. Mean, standard deviation (SD), median, interquartile range, minimum and maximum were computed for the continuous variable of age. Counts and proportions were computed for categorical variables such as gender and adverse events.

#### 4.9.2. Biomarker and Phage Statistical Analyses

Statistical analyses were performed using GraphPad Prism (version 10.2.3). The biomarkers were each analyzed using a two-way ANOVA followed by Tukey’s multiple comparisons test. The phage recovered from stool samples on day 8 were analyzed with a one-tailed unpaired *t* test with Welch’s correction.

#### 4.9.3. Microbiome Statistical Analyses

Statistical analyses were performed using R (version 4.1.2). The R package car was used to compute Levene’s tests for homogeneity of variance analysis across participants and timepoints. The R package stats was used to perform the Shapiro–Wilk test of normality and to compute *t*-tests. The R package phyloseq was used for analysis of the microbial community data and to calculate the Shannon diversity for each sample. Shannon diversity was compared between each timepoint across participants using paired *t*-tests, and the percent Shannon diversity was compared between participants at each timepoint using *t*-tests. Principal coordinates analysis (PCoA) using Bray-Curtis dissimilarity was performed to assess the beta diversity. Permutational multivariate analysis of variance (PERMANOVA) was conducted to test whether the bacterial communities sequenced have different centroids based on assignment treatment. To determine whether assumptions are met for PERMANOVA, a test of heterogeneity (ensure homogenous dispersion) was performed. The microbiota composition calculations were performed on filtered data that contained samples with a minimum of 2,400 reads, had singletons removed, each ASV was prevalent in at least 20% of samples, and taxa with less than 1% relative abundance were removed. Microbiota changes focusing on relative abundances of bacterial taxa were compared using *t*-tests and LEfSe (Linear discriminant analysis Effect Size) computed using the R package microbiomeMarker. Differences between baseline (day 1) and the end of treatment (day 8) were compared using *t*-tests, and differences across the entire study period were evaluated using LEfSe [54].

## Figures and Tables

**Table 1:** Participant demographics.

Subject Demographics	Total (*n* = 10)	ShigActive (*n* = 8)	Placebo (*n* = 2)
**Gender, *n* (%)**			
Male	5 (50.0)	4 (50.0)	1 (50.0)
Female	5 (50.0)	4 (50.0)	1 (50.0)
**Ethnicity, *n* (%)**			
Non-Hispanic	9 (90.0)	7 (87.5)	2 (100.0)
Hispanic or Latino	1 (10.0)	1 (12.5)	-
**Race, *n* (%)**			
American Indian/Alaska Native	1 (10.0)	-	1 (50.0)
Black/African American	1 (10.0)	1 (12.5)	-
White	8 (80.0)	7 (87.5)	1 (50.0)
**Age (year)**			
Mean (SD)	32.9 (6.74)	32.5 (7.59)	34.4 (0.28)
Median [Q1, Q3]	34.4 [25.5, 38.1]	32.8 [25.4, 39]	34.4 [34.2, 34.6]
Min, Max	23.7, 42.1	23.7, 42.1	34.2, 34.6

**Table 2:** Summary of adverse events by grade and treatment group.

	All Subjects (*n* = 10)		ShigActive™ (*n* = 8)		Placebo (*n* = 2)	
	No. AEs (% Total)	No. Subjects (% Total)	No. AEs (% Total)	No. Subjects (% Total)	No. AEs (% Total)	No. Subjects (% Total)
**Any Adverse Event**	9 (100)	4 (40)	9 (100)	4 (50)	-	-
Mild (Grade 1)	8 (88.9)	4 (40)	8 (88.9)	4 (50)	-	-
Moderate (Grade 2)	1 (11.1)	1 (10)	1 (11.1)	1 (12.5)	-	-
Severe (Grade 3)	-	-	-	-	-	-
Treatment-emergent *	8 (88.9)	4 (40)	8 (88.9)	4 (50)	-	-
**Any Serious Adverse Event**	-	-	-	-	-	-
**Solicited Adverse Events (Day 1–7)**	6 (100)	3 (30)	6 (100)	3 (37.5)	-	-
Diarrhea	-	-	-	-	-	-
Fever	-	-	-	-	-	-
Vomiting	-	-	-	-	-	-
Nausea	-	-	-	-	-	-
Abdominal Pain/Cramping	1 (16.7)	1 (10)	1 (16.7)	1 (12.5)	-	-
Bloating	3 (50)	2 (20)	3 (50)	2 (25)	-	-
Tenesmus	-	-	-	-	-	-
Anorexia/Loss of Appetite	-	-	-	-	-	-
Excessive Gas	2 (33.3)	2 (20)	2 (33.3)	2 (25)	-	-
Headache	-	-	-	-	-	-
**Unsolicited Adverse Events** **(Day 1–29; listed by MedDRA SOC)**	3 (100)	3 (30)	3 (100)	3 (37.5)	-	-
General disorders and administration site conditions	1 (33.3) ^†^	1 (10) ^†^	1 (33.3) ^†^	1 (12.5) ^†^	-	-
Gastrointestinal	2 (66.7) ^‡^	2 (20) ^‡^	2 (66.7) ^‡^	2 (25) ^‡^	-	-
Treatment-emergent *	2 (66.7)	2 (20)	2 (66.7)	2 (25)	-	-
**Event of Special Interest ^§^**	1	1 (10)	1	1 (12.5)	-	-

* AE causality was deemed as “related” or “non-related” to treatment per the Investigator’s discretion; however, as ShigActive™ and placebo were co-administered with sodium bicarbonate, which has known side effects, all “related” AEs were classified as treatment-emergent as they emerged during or after treatment. ^†^ 1 participant (ID# 8) experienced moderate fatigue on day 4; deemed related to treatment. ^‡^ 1 participant (ID# 15) experienced mild diarrhea on day 28 and deemed related to treatment; 1 participant (ID# 24) experienced mild constipation on day 9 and deemed not-related to treatment. ^§^ 1 participant reported pregnancy on day 90; per protocol, pregnancy is not considered an AE; time of conception determined to be prior to enrollment despite reported dual-conception methods and negative urine pregnancy testing at screening and on day 1 prior to the first dose; pregnancy was deemed not related to treatment; the participant experienced no AEs during study, and pregnancy was followed through delivery with a cesarean section ~3 weeks prior to due date due to previous preeclampsia complications with a different child, with a final outcome of baby and mother reported healthy. SOC, system organ class.

## Data Availability

The original contributions presented in the study are included in the article/Supplementary Materials; further inquiries can be directed to the corresponding author/s. All of the raw sequencing reads from this study have been uploaded to GenBank (accession number PRJNA1124105) and are accessible here: https://www.ncbi.nlm.nih.gov/bioproject/PRJNA1124105 (accessed on 4 September 2024).

## Supplementary Materials

The following supporting information can be downloaded at: https://www.mdpi.com/article/10.3390/antibiotics13090858/s1, Figure S1: Longitudinal taxonomic differences observed across the individual participants and treatment groups from day 1 (baseline) through day 29; Table S1: Clinical Blood Laboratory Values for All Subjects Throughout the Study; Table S2: Phage Quantitation in Stool in All Subjects Throughout the Study Using Spot Assays; Table S3: Phage Quantitation in Stool in All Subjects Throughout the Study Using qPCR; Table S4: Abundance Levels for *Escherichia*/*Shigella* Below the 1% Threshold; Table S5: Description of qPCR primer sequences, expected amplicon size, and amplicon peak melting temperature.
