# Supplementary material for: Safety and Tolerability of ShigActive™, a Shigella spp. Targeting Bacteriophage Preparation, in a Phase 1 Randomized, Double-Blind, Controlled Clinical Trial
Source: Antibiotics (Basel). 2024 Sep 7;13(9):858. doi: 10.3390/antibiotics13090858 (PMC11429168; doi:10.3390/antibiotics13090858)
Supplement: Supplementary file 1 [file antibiotics-13-00858-s001.zip › antibiotics-3101496-supplementary.pdf]

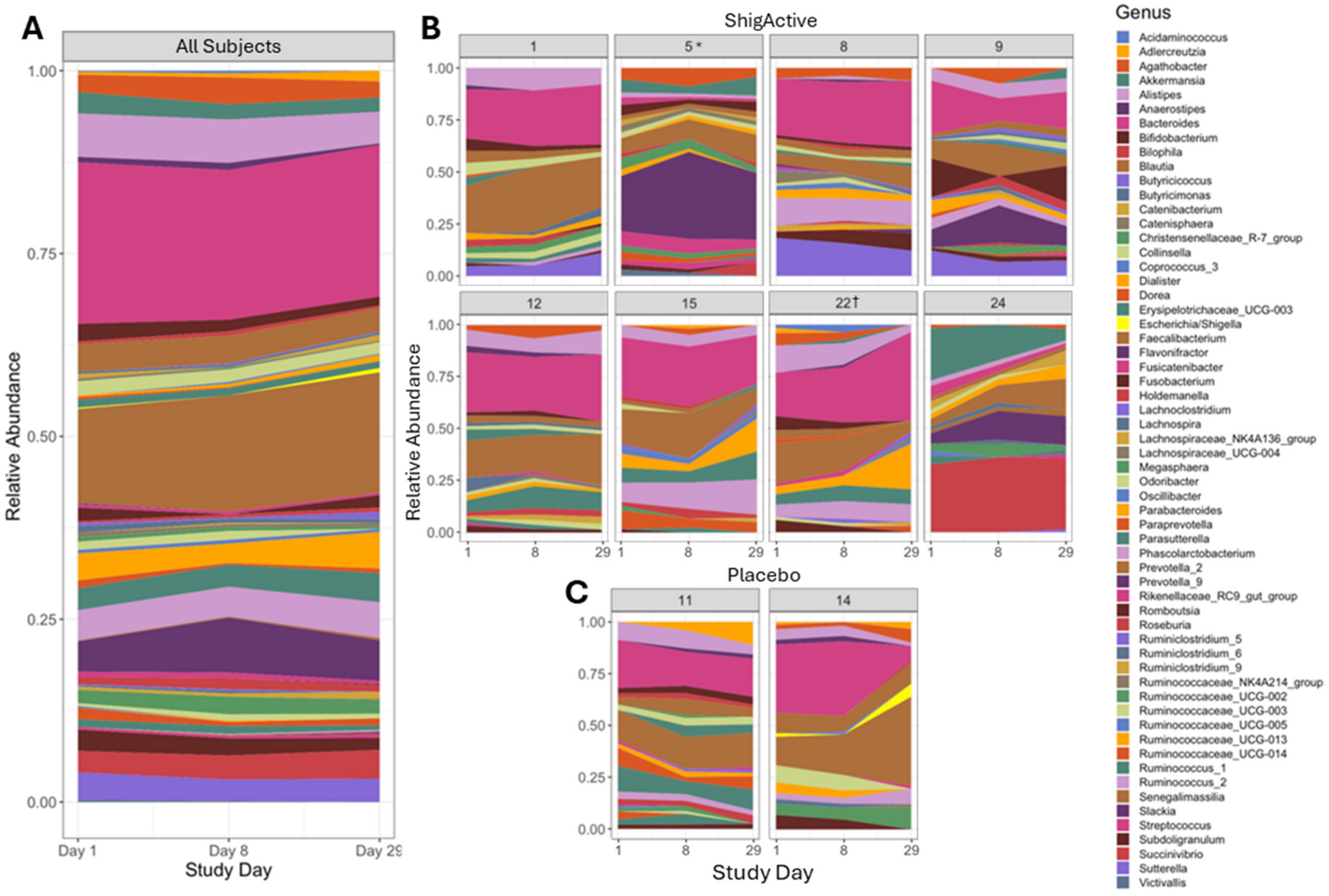

Figure S1. Longitudinal taxonomic differences observed across the individual participants and treatment groups from day 1 (baseline) through day 29. The average relative abundance for each bacterial genus from day 1 (baseline) through day 29 for (**A**) all subjects, or individual subjects in the (**B**) ShigActive™- or (**C**) placebo-treated groups. Only genera with relative abundance that are greater than 1% are shown. *Subject ID# 5 had detectable *Shigella*-specific phage through day 90. †Subject ID# 22 had no detectable phage at any timepoint.

.

Table S1. Clinical Blood Laboratory Values for All Subjects Throughout the Study.

Treat	ID	Gend	Study	Clinical Laboratory Tests							Clinical Laboratory Tests (Screening Only)				
				Hb (g/dL)	WBC (cells/mm^3)	PLT (cells/mm^3)	ANC (cells/µL)	CREAT (mg/dL)	TBIL (mg/dL)	ALT (U/L)	TLC (cells/mm^3)	Serum IgA (mg/dL)	HBsAg	HCV	HIV
Placebo	11	M	SC	15.2	3900	169000	2140	1.03	1	19	1300	96	Neg	Neg	Neg
			1	15	4390	185000	2040	1.03	0.8	18	ND	ND	ND	ND	ND
			8	14.7	***3460***	177000	2040	1	0.9	17	ND	ND	ND	ND	ND
			29	15.1	4000	190000	1840	0.99	0.6	18	ND	ND	ND	ND	ND
	14	F	SC	12.2	6890	198000	5640	0.71	0.3	11	800	229	Neg	Neg	Neg
			1	12	5270	286000	3720	0.69	0.3	10	ND	ND	ND	ND	ND
			8	**11.7**	5150	199000	3820	0.77	0.4	10	ND	ND	ND	ND	ND
			29	**11.8**	4390	253000	2860	0.73	0.5	17	ND	ND	ND	ND	ND
ShigActive™	1	M	SC	15.9	4100	241000	2140	0.97	0.6	27	1610	278	Neg	Neg	Neg
			1	15.2	3950	233000	1980	0.99	0.5	31	ND	ND	ND	ND	ND
			8	15.1	4360	233000	2350	1.02	0.5	22	ND	ND	ND	ND	ND
			29	15.3	4430	233000	2220	1.07	0.4	19	ND	ND	ND	ND	ND
	5	F	SC	13	8910	296000	5970	0.7	0.7	11	1900	259	Neg	Neg	Neg
			1	13.4	8350	323000	4830	0.81	0.5	13	ND	ND	ND	ND	ND
			8	12.1	9040	278000	6230	0.72	0.5	12	ND	ND	ND	ND	ND
			29	12.3	7820	316000	4680	0.74	0.5	11	ND	ND	ND	ND	ND
	8	M	SC	14.2	5410	285000	2930	1.14	0.7	15	1930	408	Neg	Neg	Neg
			1	14.2	5680	265000	3090	1.08	0.4	14	ND	ND	ND	ND	ND
			8	14.6	6030	294000	3940	1.15	0.7	17	ND	ND	ND	ND	ND
			29	14.2	5980	268000	3510	1.04	0.8	15	ND	ND	ND	ND	ND
	9	F	SC	13.4	4490	350000	3200	0.7	0.6	8	1020	64	Neg	Neg	Neg
			1	12.8	7630	***425000***	6100	0.73	0.4	7	ND	ND	ND	ND	ND
			8	12.6	5370	390000	4150	0.74	0.5	***6***	ND	ND	ND	ND	ND
			29	12.9	5670	319000	4110	0.74	0.7	10	ND	ND	ND	ND	ND
	12	F	SC	14.2	7390	282000	4580	0.9	0.6	11	2040	186	Neg	Neg	Neg
			1	13.8	5610	230000	3620	0.9	0.7	13	ND	ND	ND	ND	ND
			8	13.8	5150	229000	2800	0.93	0.5	11	ND	ND	ND	ND	ND
			29	14	5710	251000	3670	0.89	0.8	15	ND	ND	ND	ND	ND
	15	M	SC	13.8	7130	201000	4780	0.99	0.4	12	1700	374	Neg	Neg	Neg
			1	13.6	4970	206000	3080	1.07	0.5	15	ND	ND	ND	ND	ND
			8	**13.1**	4560	197000	2900	1.08	0.6	37	ND	ND	ND	ND	ND
			29	13.8	4540	221000	2500	1.02	0.8	16	ND	ND	ND	ND	ND

Treat	ID	Gend	Study	Clinical Laboratory Tests							Clinical Laboratory Tests (Screening Only)				
				Hb (g/dL)	WBC (cells/mm³)	PLT (cells/mm³)	ANC (cells/µL)	CREAT (mg/dL)	TBIL (mg/dL)	ALT (U/L)	TLC (cells/mm³)	Serum IgA (mg/dL)	HBsAg	HCV	HIV
	22	M	SC	16.5	6490	294000	3990	1.1	0.8	15	1550	136	Neg	Neg	Neg
			1	16.1	6510	273000	4060	1.11	0.6	10	ND	ND	ND	ND	ND
			8	17	6700	210000	4250	1.07	0.5	14	ND	ND	ND	ND	ND
			29	17.1	5850	297000	3210	1.13	0.5	13	ND	ND	ND	ND	ND
	24	F	SC	12.5	6370	271000	4320	0.81	0.7	13	1360	299	Neg	Neg	Neg
			1	12.4	5250	247000	3160	0.76	0.5	8	ND	ND	ND	ND	ND
			8	12.6	7370	252000	5360	0.76	0.6	9	ND	ND	ND	ND	ND
			29	11.9	4860	285000	2740	0.79	0.5	7	ND	ND	ND	ND	ND

ANC, absolute neutrophil count; **ALT**, alanine aminotransferase; **CREAT**, creatinine; **Hb**, hemoglobin; **HBsAg**, hepatitis B surface antigen; **HCV**, hepatitis C virus; **HIV**, human immunodeficiency virus; **IgA**, immunoglobulin A; **ND**, not determined; **Neg**, negative; **PLT**, platelet count; **SC**, screening; **TBIL**, total bilirubin; **TLC**, total lymphocyte count; **WBC**, white blood cell count.

RED text/yellow shading: abnormal, Grade 1 (mild) value; **BOLD**/*italicized*: value outside reference range, deemed not clinically significant.

REFERENCE RANGES:

Hb: Male: 13.5-17.5 g/dL; Female: 12.0-16.0 g/dL
WBC: 3,500-11,500 /mm³
PLT: 140,000-400,000 /mm³
ANC: 1,560-8,100 /uL
CREAT: Male: 0.7-1.3 mg/dL; Female: 0.6-1.2 mg/dL
TBIL: 0.2-1.2 mg/dL
ALT: 7-45 U/L

Table S2. Phage Quantitation in Stool in All Subjects Throughout the Study Using Spot Assays.

Treatment	ID	Total PFU/g Stool Day 1	Day 8	Day 15	Day 29	Day 90
Placebo	11	0	0	0	0	0
	14	0	0	0	0	0
ShigActive™	1	0	5.05E+06*	0	0	0
	5	0	4.60E+05*	3.30E+06*	4.20E+04§	2.15E+04‖
	8	0	2.00E+03†	0	0	0
	9	0	3.75E+05*	0	0	0
	12	0	5.75E+04‡	0	0	0
	15	0	6.10E+04‖	0	0	0
	22	0	0	0	0	0
	24	0	5.95E+04*	0	0	0

For comparison, superscript indicates ShigActive™ monophages detected by qPCR (Table S3):
* all 5 monophages (A-E); †2 monophages (B & C); ‡ 3 monophages (A, B, C & E); § 1 monophage (D); and ‖ no detectable monophages.

Table S3. Phage Quantitation in Stool in All Subjects Throughout the Study Using qPCR.

Treatment	ID#	Sample	Replicate	Ct Value for Each ShigActive™ Monophage A	B	C	D	E
Placebo	11	*ShigActive, (+) control*	*1*	***14.1***	***12.8***	***11.2***	***12.3***	***14.5***
		Day 1	1	-	-	-	-	-
			2	-	38.0	-	-	-
			3	-	-	-	-	35.0
		Day 8	1	-	-	-	-	-
			2	-	-	-	-	-
			3	-	-	-	-	-
		Day 15	1	-	-	-	-	-
			2	-	-	-	-	-
			3	-	-	-	-	-
		Day 29	1	-	-	-	-	37.5
			2	-	35.7	-	-	-
			3	37.9	-	-	-	-
		Day 90	1	-	-	-	-	-
			2	-	-	-	-	-
			3	-	-	-	-	-
		Water, (-) control	*1*	-	-	-	-	-
Placebo	14	*ShigActive, (+) control*	*1*	***14.0***	***14.2***	***11.2***	***12.5***	***14.8***
		Day 1	1	-	36.5	-	-	-

Treatment	ID#	Sample	Replicate	Ct Value for Each ShigActive™ Monophage				
				A	B	C	D	E
			2	-	-	-	-	-
			3	-	-	-	-	-
		Day 8	1	-	-	-	-	-
			2	-	-	-	-	-
			3	-	-	-	-	-
		Day 15	1	-	-	-	-	-
			2	-	-	-	-	-
			3	-	-	-	-	-
		Day 29	1	-	-	-	-	-
			2	-	-	38.1	-	-
			3	-	-	-	-	-
		Day 90	1	-	-	-	-	-
			2	-	-	-	-	-
			3	-	-	-	-	-
		Water, (-) control	*1*	-	-	-	-	-
ShigActive™	1	*ShigActive, (+) control*	*1*	*13.7*	*13.1*	*12.1*	*11.5*	*13.8*
		Day 1	1	-	-	-	-	-
			2	-	-	-	-	-
			3	-	-	-	-	-
		Day 8	1	26.8	23.4	23.2	25.9	26.4
			2	**26.7**	**23.4**	**22.9**	**25.9**	**26.3**
			3	**26.6**	**23.7**	**22.9**	**26.0**	**26.2**
		Day 15	1	-	-	-	-	-
			2	-	-	-	-	-
			3	-	-	-	-	-
		Day 29	1	-	-	-	-	-
			2	-	-	-	-	-
			3	-	-	-	-	37.8
		Day 90	1	-	-	-	-	-
			2	-	-	-	-	-
			3	-	-	-	-	-
		Water, (-) control	*1*	-	-	-	-	-
ShigActive™	5	*ShigActive, (+) control*	*1*	***13.3***	***12.3***	***13.3***	***12.7***	***14.3***
		Day 1	1	-	-	-	-	-
			2	-	37.5	-	-	-
			3	-	-	-	-	-
		Day 8	1	27.1	23.3	23.8	27.2	27.2
			2	**27.2**	**23.4**	**23.7**	**27.1**	**27.3**
			3	**27.1**	**23.6**	**23.8**	**27.2**	**27.2**
		Day 15	1	**32.6**	**28.5**	**30.5**	**28.6**	**24.0**
			2	**33.6**	**28.9**	**31.5**	**29.1**	**24.3**
			3	**32.4**	**28.8**	**31.6**	**28.4**	**24.3**

Treatment	ID#	Sample	Replicate	Ct Value for Each ShigActive™ Monophage				
				A	B	C	D	E
		Day 29	1	-	-	-	**29.2**	-
			2	-	-	-	**29.2**	39.1
			3	-	-	-	**28.7**	
		Day 90	1	-	-	-	-	-
			2	-	-	-	-	-
			3	-	34.6	-	-	37.0
		Water, (-) control	*1*	-	-	-	-	-
ShigActive™	8	*ShigActive, (+) control*	*1*	*14.4*	*10.9*	*11.4*	*12.2*	*15.6*
		Day 1	1	-	-	-	-	-
			2	-	-	-	-	-
			3	-	-	-	-	-
		Day 8	1	-	33.0	32.7	-	-
			2	-	**31.1**	**31.0**	-	-
			3	-	36.2	**30.8**	-	-
		Day 15	1	-	-	-	-	-
			2	-	-	-	-	-
			3	-	-	-	-	-
		Day 29	1	-	-	-	-	-
			2	-	-	-	-	-
			3	-	-	-	-	-
		Day 90	1	-	-	-	-	-
			2	-	-	-	-	-
			3	-	-	-	-	-
		Water, (-) control	*1*	-	-	-	-	-
ShigActive™	9	*ShigActive, (+) control*	*1*	*13.5*	*12.8*	*12.7*	*12.8*	*14.8*
		Day 1	1	-	-	-	-	-
			2	-	-	-	-	-
			3	-	-	-	-	-
		Day 8	1	28.9	24.9	25.7	29.4	29.3
			2	**28.7**	**25.0**	**25.6**	**29.9**	**29.3**
			3	**28.7**	**24.8**	**25.5**	**29.6**	**29.7**
		Day 15	1	-	-	-	-	-
			2	-	-	-	-	-
			3	-	-	-	-	-
		Day 29	1	-	-	-	-	35.0
			2	-	-	-	-	-
			3	-	-	-	-	36.1
		Day 90	1	-	-	-	-	-
			2	-	-	-	-	-
			3	-	-	-	-	-
		Water, (-) control	*1*	-	-	-	-	-
ShigActive™	12	*ShigActive, (+) control*	*1*	*12.7*	*12.2*	*12.0*	*14.1*	*14.4*

Treatment	ID#	Sample	Replicate	Ct Value for Each ShigActive™ Monophage				
				A	B	C	D	E
		Day 1	1	-	-	38.0	-	-
			2	-	38.0	-	36.3	-
			3	-	-	-	-	-
		Day 8	1	32.4	27.2	28.3	35.4	31.9
			2	**31.9**	**27.6**	**28.8**	36.2	**31.5**
			3	**30.8**	**27.5**	**28.0**	38.4	**29.9**
		Day 15	1	-	-	-	-	-
			2	-	-	-	-	-
			3	-	-	-	-	-
		Day 29	1	-	-	-	-	-
			2	-	-	-	-	-
			3	-	-	-	-	-
		Day 90	1	-	-	-	-	-
			2	-	-	-	-	-
			3	-	-	-	-	-
		Water, (-) control	*1*	-	-	-	-	-
ShigActive™	15	*ShigActive, (+) control*	*1*	*15.0*	*14.4*	*11.3*	*12.7*	*12.6*
		Day 1	1	-	-	37.9	-	-
			2	-	-	39.4	-	-
			3	-	-	-	-	-
		Day 8	1	-	-	-	-	34.8
			2	-	-	-	-	34.6
			3	-	-	-	-	36.1
		Day 15	1	-	-	-	-	-
			2	-	-	-	-	-
			3	-	-	-	-	-
		Day 29	1	-	-	-	-	-
			2	-	-	-	-	-
			3	-	-	-	-	-
		Day 90	1	-	-	-	-	-
			2	-	-	-	-	-
			3	-	-	-	-	-
		Water, (-) control	*1*	-	-	-	-	-
ShigActive™	22	*ShigActive, (+) control*	*1*	*14.3*	*12.9*	*11.6*	*14.2*	*15.5*
		Day 1	1	-	-	-	-	-
			2	-	-	-	-	-
			3	-	-	-	-	-
		Day 8	1	-	-	-	-	-
			2	-	-	-	-	-
			3	-	-	-	-	-
		Day 15	1	-	-	-	-	-
			2	-	-	-	-	-

Treatment	ID#	Sample	Replicate	Ct Value for Each ShigActive™ Monophage				
				A	B	C	D	E
			3	-	33.8	-	-	-
		Day 29	1	-	-	-	-	-
			2	-	35.8	-	-	-
			3	-	-	-	-	-
		Day 90	1	-	-	-	-	-
			2	-	-	-	-	-
			3	-	-	-	-	-
		Water, (-) control	*1*	-	-	-	-	-
ShigActive™	24	*ShigActive, (+) control*	*1*	*13.3*	*13.5*	*11.9*	*13.3*	*14.9*
		Day 1	1	-	-	-	-	-
			2	-	-	-	-	-
			3	-	-	-	-	-
		Day 8	1	29.7	27.1	25.8	30.0	30.9
			2	**29.8**	**26.9**	**25.8**	**30.2**	**30.9**
			3	**29.5**	**27.0**	**25.7**	**30.4**	**30.4**
		Day 15	1	34.9	**32.9**	**31.5**	-	-
			2	33.8	**31.3**	**31.1**	-	-
			3	-	**31.9**	-	-	34.0
		Day 29	1	-	36.0	-	-	-
			2	-	-	37.9	-	-
			3	-	-	-	-	-
		Day 90	1	-	-	-	-	-
			2	-	-	-	-	-
			3	-	-	-	-	-
		Water, (-) control	*1*	-	-	-	-	-
Spiked Control		ID# 11, Day 1	1	**23.7**	**23.5**	**25.1**	**23.9**	**24.8**
		ID# 14, Day 1	1	**24.6**	**25.1**	**24.4**	**24.0**	**25.5**
		ID# 1, Day 1	1	24.5	23.7	24.9	24.5	25.1
		ID# 5, Day 1	1	**24.1**	**24.1**	**31.1**	**24.5**	**26.3**
		ID# 8, Day 1	1	**23.7**	**23.7**	**28.4**	**24.6**	**25.3**
		ID# 9, Day 1	1	**23.8**	**23.8**	**25.8**	**25.3**	**25.0**
		ID# 12, Day 1	1	**24.9**	**26.1**	**25.8**	**27.4**	**28.7**
		ID# 15, Day 1	1	**24.9**	**24.5**	**31.0**	**25.2**	**26.5**
		ID# 22, Day 1	1	**24.0**	**24.3**	**30.6**	**24.2**	**26.5**
		ID# 24, Day 1	1	**24.1**	**23.8**	**27.2**	**24.2**	**25.5**
		ShigActive™ 1x10^{7} PFU/mL, control	*1*	***22.4***	***22.3***	***21.2***	***24.1***	***24.7***
		ShigActive™, (+) control	*1*	***13.2***	***12.8***	***12.3***	***13.1***	***14.2***
		Water, (-) control	*1*	-	-	-	-	-

Treatment	ID#	Sample	Replicate	Ct Value for Each ShigActive™ Monophage				
				A	B	C	D	E

Italics, controls where no stool extract was added to the reaction; Water, (-) control: negative control using water as test article; ShigActive, (+) control: positive control using only ShigActive™ at 1x10^{10} PFU/mL as test article; ShigActive™ 1x10^{7} PFU/mL control: positive control for spiked samples using only ShigActive™ at 1x10^{7} PFU/mL as test article.

"-", no detectable ShigActive™ phage in sample determined by absence of qPCR product with the expected melting temperature (Table **S5**).

Green, samples were scored as positive for detection of the indicated monophage if the Ct value was >10 and <33.

Blue shading, stool extracts from the indicated study day from ShigActive™-treated subjects.

Gray shading, stool extracts from the indicated study day from placebo-treated subjects.

Table S4. Abundance Levels for *Escherichia*/*Shigella* Below the 1% Threshold.

Treatment	ID	Relative Abundance		
		Day 1	Day 8	Day 29
Placebo	11	2.9116000E-04	1.4004300E-04	4.7480000E-04
	14	1.2950639E-02	9.9530000E-04	4.6633081E-02
ShigActive™	1*	0.0000000E+00	0.0000000E+00	0.0000000E+00
	5†	8.4543300E-04	1.3750400E-04	0.0000000E+00
	8	9.0707200E-04	3.0933000E-04	7.2248245E-05
	9*	0.0000000E+00	0.0000000E+00	0.0000000E+00
	12	2.0464680E-03	5.1654090E-03	4.0317600E-03
	15	1.0203500E-04	4.1965672E-05	0.0000000E+00
	22‡	2.0827900E-04	0.0000000E+00	0.0000000E+00
	24	9.0978565E-05	3.8121500E-04	1.1701400E-04

* No *Escherichia*/*Shigella* detected at any timepoint;
† Subject with detectable levels of *Shigella*-specific phage in stool out through day 90 by spot assay;
‡ Subject with no detectable levels of *Shigella*- or ShigActive™-specific phage in stool at any timepoint by either spot assay or qPCR, respectively.
Yellow shading, taxa levels decreased over time.

Table S5. Description of qPCR primer sequences, expected amplicon size, and amplicon peak melting temperature.

Phage Target	Forward Primer (5′-3′)	Reverse Primer (5′-3′)	Amplicon Size (bp)	Peak Melt Temp (°C)
A	TCG CAA CGA TAT AAG GAC CC	CGC TTT GCA GCT TTA ATT CC	116	74.7
B	TGC ACG AGTCAA AAG GTC AG	GTG AAG GCG AGT TTG TGG TT	180	77.4
C	ACA AGG CTA GAA TGC GCC TA	AAT ACA GTTTCA CCG GAC GC	232	80.7
D	CGT TGC CAA CTT AAA ACT GCA CCC	CAT CGA AAA GGG TCA GGA GCA TAT	271	78.6
E	CCA GGA TGG CAT TGA ACT CT	GGT GAA TTT GCT TCG TGG AT	121	77.0
